# Assistive Technologies in Dementia Care: An Updated Analysis of the Literature

**DOI:** 10.3389/fpsyg.2021.644587

**Published:** 2021-03-24

**Authors:** Alessandro Pappadà, Rabih Chattat, Ilaria Chirico, Marco Valente, Giovanni Ottoboni

**Affiliations:** ^1^Department of Psychology, University of Bologna, Bologna, Italy; ^2^“G. Prodi” Interdipartimental Center for Cancer Research, Bologna, Italy

**Keywords:** dementia, technology-assistive/supportive, COVID-19 pandemic, quality of life, caregivers, psychology

## Abstract

**Objectives:** Technology can assist and support both people with dementia (PWD) and caregivers. Recently, technology has begun to embed remote components. Timely with respect to the pandemic, the present work reviews the most recent literature on technology in dementia contexts together with the newest studies about technological support published until October 2020. The final aim is to provide a synthesis of the timeliest evidence upon which clinical and non-clinical decision-makers can rely to make choices about technology in the case of further pandemic waves.

**Methods:** A review of reviews was performed alongside a review of the studies run during the first pandemic wave. PsycInfo, CINAHL, and PubMed-online were the databases inspected for relevant papers published from January 2010.

**Results:** The search identified 420 articles, 30 of which were reviews and nine of which were new studies meeting the inclusion criteria. Studies were first sorted according to the target population, then summarized thematically in a narrative synthesis. The studies targeting technologies for PWD were categorized as follows: monitoring and security purposes, sustaining daily life, and therapeutic interventions. Each category showed potential benefits. Differently, the interventions for caregivers were classified as informative, psycho-education programs, psychosocial-supportive, therapeutic, and cognitive/physical training. Benefits to mental health, skills learning, and social aspects emerged.

**Conclusions:** The evidence shows that technology is well-accepted and can support PWD and caregivers to bypass physical and environmental problems both during regular times and during future pandemic waves. Nevertheless, the lack of a common methodological background is revealed by this analysis. Further and more standardized research is necessary to improve the implementation of technologies in everyday life while respecting the necessary personalization.

## Introduction

According to recent statistics, a demographic revolution is currently underway: the average life expectancy is rising worldwide, and the population of persons aged over 60 is going to continually grow until 2050 (United Nations, Department of Economic and Social Affairs, and Population Division, 2020; WHO | Global Action Plan on the Public Health Response to Dementia 2017–2025, 2020). Even though aging is part of human development, one of the main risks associated with it concerns dementia: 5% of the world population aged over 65 is affected by some types of dementia, and this prevalence doubles around every 5 years (Corrada et al., 2010).

Recently, assistive technologies (ATs) have become one of the fundamental pillars of health strategies. They include “*any product or technology-based service that enables people of all ages with activity limitations in their daily life, education, work or leisure*” (Association for the Advancement of Assistive Technology in Europe [WorldCat Identities], 2018). Regarding dementia, ATs can increase motor autonomy and reduce the risks associated with wandering thanks to their GPS technology (Liu et al., 2017); they can also sustain people's cognitive abilities—those required to accomplish necessary daily activities (Nishiura et al., 2019). Again, ATs play a role in supporting the policies surrounding *aging in place*. Indeed, they can delay people's institutionalization or reduce the number of severe clinical cases requiring admission to care homes (Brittain et al., 2010). Besides, technology is useful when admission into a care facility becomes mandatory or is the person's preferred option. In these cases, ATs allow for easier communication between residents and relatives and overcome social barriers (Winstead et al., 2013).

Furthermore, ATs increase people's safety by sustaining independence while respecting dignity (Brittain et al., 2010). Finally, ATs are associated with benefits when conveying rehabilitation and psychosocial interventions (Peek et al., 2014). Technological devices are cheap and affordable (Al-Oraibi et al., 2012). Intuitive interfaces support the users' sense of control by highlighting cause–effect relations between tasks and actions (Leng et al., 2014). Thanks to personalized items and control processes focused on responding to specific needs and preserving abilities, they increase users' participation in interventions (Smith and Mountain, 2012; Darcy et al., 2017). Finally, technology promotes remote support and assistance by overcoming environmental barriers (Azad et al., 2012).

On the other hand, ATs often present a few limitations. Devices might be experienced as intrusive and invading the users' privacy (Dorsten et al., 2009). Again, they can be obstructive and increase the stigma that sometimes comes with the disease (Pritchard and Brittain, 2015). Moreover, complicated features, or intense learning sessions, might underline cognitive abilities loss, leading to frustration and rejection of technology (Peek et al., 2014).

Recently, the remote feature has begun to characterize ATs more and more, as it becomes useful to bridge the distance between people (Cuffaro et al., 2020). Capitalizing on the positive evidence about AT and dementia (Meiland et al., 2017), it may be conceivable to assume that ATs might play a key role in the attempts to alleviate the future burden lockdowns might bring with them (INDUCT, 2020). At the same time, to keep people safe, technological devices might support people with dementia and caregivers during the months of lockdown (Meiland et al., 2020).

Timely with respect to the pandemic, the present work reviews the most recent literature reviews on ATs in dementia contexts together with a review of the new studies adopting ATs during the virus outbreak.

The current work's final aim is to provide a tangible summary upon which clinical and non-clinical decision-makers can base their choices about which technological intervention tools they can deploy to directly compensate/improve specific dysfunctionalities affecting either people with dementia or caregivers even during future pandemic waves.

## Method

### Data Collection and Strings Definition

PsycINFO, PubMed, and CINAHL were the online databases where we sought peer-reviewed papers published from January 2010 to October 2020 (Table 1). The research query combined keywords from three different research strings (A, B, C) through the Boolean operators “AND” and “OR” (Table 2). String A included the studies that were related to technology in general. Due to the lack of standardized terminology (Roest et al., 2017), several terms were derived from the APA thesaurus. String B selected the target population. String C filtered for the methodology of interest.

**Table 1:** Initial search data.

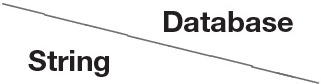	**PsycINFO**	**PubMed**	**CINAHL**
A	49,933	66,372	147,950
A AND B	354	592	1,082
A AND B AND C	109	111	197

**Table 2:** Complete query used.

**PsycINFO**	**PubMed**	**CINAHL**
(KW (technology or technologies) OR KW telemedicine OR KW assistive device OR KW App OR KW computer OR KW tablet OR KW telecommunication OR KW web-based OR KW online OR KW internet OR KW (telecare or “tele care”) OR KW (ehealth or e-health or electronic health) OR KW (telehealth or “tele health”) OR KW digital OR KW (videotelephone or “video phone”) OR KW video chat OR KW video communication)) AND (KW (dementia or alzheimer)) AND (TI intervention OR KW intervention OR AB intervention)	((((technology[Other Term] OR technologies[Other Term] OR telemedicine[Other Term] OR assistive device[Other Term] OR App[Other Term] OR computer[Other Term] OR tablet[Other Term] OR telecommunication[Other Term] OR web-based[Other Term] OR online[Other Term] OR internet[Other Term] OR telecare[Other Term] OR tele care[Other Term] OR ehealth[Other Term] OR e-health[Other Term] OR electronic health[Other Term] OR telehealth[Other Term] OR tele health[Other Term] OR digital[Other Term] OR videotelephone[Other Term] OR video phone[Other Term] OR video chat[Other Term] OR video communication[Other Term])) AND (dementia[Other Term] OR alzheimer[Other Term]))) AND ((intervention[Title/Abstract]) OR intervention[Other Term])	((SU (technology or technologies) OR SU telemedicine OR SU assistive device OR SU App OR SU computer OR SU tablet OR SU telecommunication OR SU web-based OR SU online OR SU internet OR SU (telecare or “tele care”) OR SU (ehealth or e-health or electronic health) OR SU (telehealth or “tele health”) OR SU digital OR SU (videotelephone or “video phone”) OR SU video chat OR SU video communication)) AND SU (dementia or alzheimer))) AND (TI intervention OR SU intervention OR AB intervention)

#### Typology of Review and Eligibility Criteria

To summarize the most recent literature related to the use of technology, the present review combines the review of reviews methodology (Smith et al., 2011) with a literature review including the most recent studies on the topic that are still not reviewed.

##### Inclusion Criteria

The included studies are those that are as follows: peer-reviewed, published from January 2010 to October 2020, available in English or Italian, and those that deal with any technological devices. The studies analyze interventions on both people diagnosed with dementia and their caregivers. Moreover, we ascertain any method: experimental, quasi-experimental, or single-case studies.

##### Exclusion Criteria

We excluded studies if the target population was composed of MCI or the authors did not explicitly sort the results between PWD and MCI. Moreover, we do not accept any papers reporting only dementia technological assessments or diagnoses.

#### Selection Process

**Figure 1:**
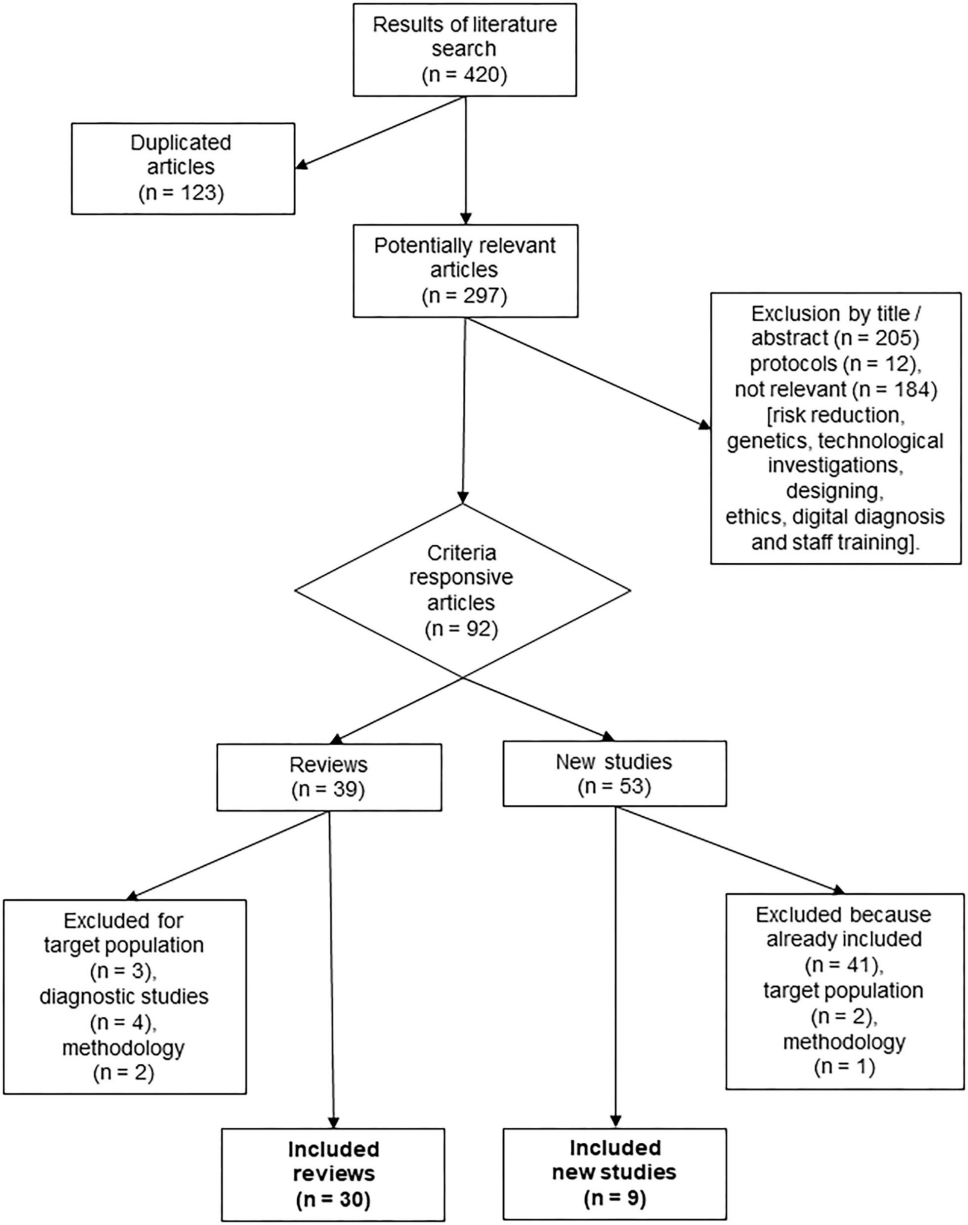
Revision flow chart.

The selection process is showed in the PRISMA flow diagram in Figure 1. The search brought out 420 papers, 123 of which were removed because of duplicates. Both the title and the abstract of the 297 remaining documents were checked. Ninety-two documents emerged from this former analysis, of which 39 were review papers, and 53 were new studies. Nine reviews were further excluded. Once they were fully read, they did not meet the eligibility criteria: three were excluded for the target population, four were excluded because devices were aimed only at diagnosing, and two were excluded for the methodology.

Out of the 53 new studies, 41 were already in the reviews, and three of them did not meet the inclusion criteria: two of them due to the target population and one for the methodology (i.e., protocol report). In the end, 30 reviews and nine new studies were included in the present work and thoroughly analyzed.

#### Stages of Analysis

At first, we assessed the quality of the included systematic reviews through either the AMSTAR tool guidance for the systematic reviews (Shea et al., 2009) or the SANRA scale for the non-systematic reviews (Baethge et al., 2019; Tables 3, 4, respectively). A formal assessment of the new studies was not performed, as it was urgent to update the pandemic-related literature, despite the study quality.

**Table 3:** Quality assessment of systematic reviews using AMSTAR.

**Review**	**A priori design**	**Duplicate study selection and data extraction**	**Comprehensive literature search**	**Publication status criteria**	**List of included and excluded studies**	**Characteristics of included studies**	**Quality assessment**	**Study quality used appropriately in formulating conclusions**	**Appropriate methods used to combine studies**	**Publication bias assessed**	**Conflict of interest stated**	**Total**
Boots et al. (2014)	Y	CA	Y	N	N	Y	Y	Y	NA	N	Y	6
Brims and Oliver (2019)	Y	N	Y	Y	N	Y	N	Y	Y	Y	Y	8
Daly Lynn et al. (2019)	Y	Y	Y	Y	N	Y	N	Y	NA	N	Y	7
Dam et al. (2016)	Y	Y	Y	Y	N	Y	Y	Y	NA	Y	Y	9
Egan et al. (2018)	Y	Y	Y	N	N	Y	Y	Y	NA	Y	Y	8
El-Saifi et al. (2018)	Y	Y	Y	Y	N	Y	N	N	NA	N	Y	6
Fleming and Sum (2014)	Y	CA	Y	N	N	Y	Y	Y	NA	N	N	5
García-Casal et al. (2017)	Y	CA	Y	N	N	Y	Y	Y	Y	N	Y	7
Godwin et al. (2013)	Y	N	Y	Y	N	Y	N	Y	NA	N	Y	6
Hopwood et al. (2018)	Y	Y	Y	Y	N	N	Y	Y	NA	Y	Y	8
Jackson et al. (2016)	Y	Y	Y	Y	N	Y	Y	Y	NA	Y	Y	9
Lazar et al. (2014)	Y	Y	Y	Y	N	Y	N	Y	NA	N	Y	7
Leng et al. (2020)	Y	Y	Y	N	N	Y	Y	Y	Y	Y	Y	9
Liapis and Harding (2017)	Y	CA	Y	Y	N	Y	Y	Y	NA	N	Y	7
Lucero et al. (2019)	Y	Y	Y	N	N	Y	Y	Y	NA	Y	Y	8
Maia et al. (2018)	Y	Y	Y	Y	N	Y	Y	Y	NA	Y	Y	9
McKechnie et al. (2014)	Y	CA	Y	Y	N	Y	Y	Y	NA	N	Y	7
Parra-Vidales et al. (2017)	Y	Y	Y	N	N	Y	N	Y	NA	N	N	5
Pinto-Bruno et al. (2017)	Y	Y	Y	N	N	Y	Y	Y	NA	N	Y	7
Ruggiano et al. (2018)	Y	Y	Y	Y	N	Y	Y	Y	NA	N	Y	8
Scott et al. (2016)	Y	Y	Y	N	N	Y	Y	Y	Y	Y	Y	9
Tyack and Camic (2017)	Y	Y	Y	Y	N	Y	Y	Y	NA	N	Y	8
Waller et al. (2017)	Y	Y	Y	Y	N	Y	Y	Y	NA	Y	Y	9
**Total yes per item**	23	16	23	14	0	22	17	22	4	10	21	

*Scores: CA, can't answer; N, no; NA, not applicable; Y, yes (Shea et al., 2009)*.

**Table 4:** Quality assessment of non-systematic reviews using SANRA.

**Review**	**Justification of the article's importance for the readership**	**Statement of concrete aims or formulation of questions**	**Description of the literature search**	**Referencing**	**Scientific reasoning**	**Appropriate presentation of data**	**Total**
Brando et al. (2017)	2	2	2	2	2	1	11
Dove and Astell (2017)	2	2	2	2	2	2	12
Klimova and Maresova (2017)	2	2	2	2	1	1	10
Lorenz et al. (2019)	1	2	1	2	2	2	10
Neubauer et al. (2018)	2	2	2	1	2	2	11
Rathnayake et al. (2019)	2	2	2	2	2	2	12
Yousaf et al. (2019)	2	2	2	2	2	2	12
**Total**	13	14	13	13	13	12	

*Scores: 0–2 (Baethge et al., 2019)*.

Moreover, we aggregated the new studies to calculate the overall risk ratio (Balduzzi et al., 2019). A risk ratio (RR) >1 signifies that the intervention groups manifest better outcomes than the control ones. The packages *meta* and *metasens* within the freely available statistical environment R facilitated ratio calculation (Schwarzer et al., 2015; R Core Team, 2019).

Yielded works were parsed according to the target population (PWD and/or caregivers). In Table 5, we classified the data from the reviews about PWD. In Table 6, we instead reported the data about the new studies (i.e., sample size, characteristics, settings, and intervention length). In Table 7, we summarized the data from the reviews about PWD caregivers. In particular, Tables 5, 7 display data about the types of conducted interventions, focus, used methodology, main results, and review conclusions. Hence, a thematic analysis of the outcomes was performed to classify the papers according to the aims underpinning the technological devices studied. Narrative synthesis integrates and appraises the quantitative and qualitative findings and the inclusion of studies using different methodologies. Two authors (AP and GO) reviewed and discussed the inclusion potential studies, and any discrepancy was resolved by a third reviewer (RC) through discussion until an agreement was reached. Thematic analysis was performed as an iterative process. Studies were read and re-read by the researchers, and key themes were identified for each paper and then amalgamated and integrated across studies.

**Table 5:** Interventions for PWD.

**References**	**Focus**	**Methods**	**Interventions**	**Results**	**Conclusions**
Brando et al. (2017)^*^	Analysis of the advantages and disadvantages associated with the implementation of technology into works with PWD and caregivers.	Literature Review. 30 studies, 27 on PWD.	Cognitive rehabilitation using technologies (videogames, VR setting, smartphone, computer and tablet). Cognitive assessment using digital tests.	Cognitive rehabilitation leads to a large generalization of the benefits. Significative outcomes on cognitive and depressive symptoms using videogames. Greater results on self-efficacy, perceived improvement, involvement and cognitive symptoms using VR rehabilitation than traditional activities. Positive effects on QoL using everyday technologies. Using digital tests for the assessment allow to standardize the administration process and the presentation of stimuli.	Cognitive rehabilitation using technologies has advantages over traditional rehabilitation. Further RCT studies are required to compare the advantages associated with different devices.
Brims and Oliver (2019)	Analysis of the effectiveness of ATs in improving the safety of PWD.	Systematic Review and Meta-analysis. Three RCTs.	Interventions using devices (sensors and tech-armbands) to increase safety in domestic setting.	The probability of a fall occurring was 50% lower in the intervention group [risk ratio 0.50 95% CI (0.32, 0.78); Z = 3.03; *p* = 0.002]. Significant fewer risky behavior in 1/3 studies (*p* < 0.001). No significant differences emerged between groups in care home admission, QoL and depressive symptoms.	Current evidence supports the use of safety AT by PwD. Further research is required to infer causality.
Daly Lynn et al. (2019)	Analysis of the ATs used for PWD in residential care settings.	Systematic Review, 61 studies.	Interventions based on technologies. Telecare (23), light therapy (4), pet robots (12), simulated presence therapy (9), leisure activities (8) and ADL (5).	Telecare technologies – improvement in safety and increased PWD's autonomy. Light therapy – Improvement in circadian rhythms. Pet robots – decreased BPSD and depressive symptoms; increased social interactions. Daily living activities – positive effects on cognition, communication and physical activity. ADL – increased autonomy in personal hygiene, decreased stress using digital prompts.	Positive outcomes support the potential of ATs in dementia context. More standardized studies are required to explore the effectiveness of each device.
Dove and Astell (2017)	Analysis of the available motion-based technologies in dementia context.	Literature Review. Thirty-one studies, section of 25/31 on PWD.	Interventions combining cognitive stimulation, physical activity and leisure activities using videogames based on motion sensors.	Motion-based technologies have benefits on general cognition, mobility, balance, fall risks, self-esteem, well-being and social health.	Motion-based technologies are feasible to stimulate PWD. A positive acceptability emerged.
El-Saifi et al. (2018)	Analysis of interventions aimed at improving medication adherence in PWD.	Systematic Review, 20 studies, one relevant.	Intervention of tele-monitoring during day/night time.	Significative compliance in the intervention group (81%), compared to the control group (66%), *p* < 0.05. Also, unmonitored patients' compliance fell of 12%.	Tele-monitoring was the only intervention able to increase PWD's compliance. Further standardized studies are required.
Fleming and Sum (2014)	Analysis of the effectiveness of ATs in dementia care.	Systematic Review, 41 studies.	Interventions based on ATs to sustain: daily living, safety, therapies and telecare.	ATs for daily life were positively evaluated, but their usage decreased over time. - Safety ATs had potential benefits, but technical issues also emerged. – Positive outcomes on BPSD and circadian rhythms came out using technology-based therapy. -Telecare led to positive outcomes concerning cognitive training and medication adherence.	Mixed results emerged using ATs in dementia care. Further standardized studies are required to assess the effectiveness of technologies for PWD
García-Casal et al. (2017)	Analysis of the effectiveness of computer-based cognitive interventions targeting PWD.	Systematic Review and Meta-analysis.	Computer-based interventions of cognitive training, cognitive rehabilitation, cognitive stimulation and cognitive recreation.	Moderate effects on cognition, assessed with MMSE and HDS-R (SMD −0.69; 95% CI = −1.02 a to 0.37; *P* < 0.0001; I∧2 = 29%); greater effects on cognition of computer-based interventions compared to traditional interventions (SMD 0.48; 95% CI = 0.09–0.87; *P* = 0.02; I∧2 = 2%). Small effects in depression, assessed with GDS and CES-D (SMD 0.47; 95% CI = 0.16 a 0.78; *P* = 0.003; I∧2 = 0%). Moderate effects on anxiety, assessed with STAI (SMD 0.55; 95% CI = 0.07 a 1.04; *P* < 0.03; I∧2 = 42%). No significant effects on IADL (*p* > 0.05).	Computer-based cognitive interventions have moderate effects on cognition and anxiety; small effects on depression. Computer-based interventions have greater effects over the traditional ones. Longer-term follow up are required to examine effects' retention.
Klimova and Maresova (2017)	Analysis of the effectiveness of computer-based cognitive training for PWD and MCI.	Mini Review, section of four RCTs on PWD.	Computer-based cognitive training interventions.	1/4 study showed improvement in episodic memory and abstract reasoning. 1/4 study was effective in delaying the progression of the cognitive impairment. 2/4 studies revealed no effects.	Mixed results emerged. Further standardized studies are required to examine the effectiveness of computer-based cognitive training on PWD.
Lazar et al. (2014)	Analysis of the use of ICTs for facilitating reminiscence therapy.	Systematic Review, 44 studies.	Reminiscence therapy using technologies (videogames, multimedia and digital interfaces).	Technologies accommodate for motor and sensor impairments, using devices as earphones, image projectors or touchscreens. ICTs allow to compensate for memory deficits. Also, ICTs facilitate the administration process using clouds or telecare.	Technologies enrich reminiscence therapy for PWD. Further studies should focus on the effectiveness at different stages of dementia.
Liapis and Harding (2017)	Analysis of the effectiveness of computer-based therapies for PWD.	Systematic Review, section of five relevant studies, one RCT.	Technology-based therapy interventions and leisure activities for PWD.	Interventions have been evaluated as feasible and enjoyable by PWD. No quantitative improvement in cognition emerged using MMSE. People with mild and moderate dementia preferred videogames; people with severe dementia preferred listening to music or watching videos.	Potential benefits emerged, but more standardized studies are required to examine the effectiveness of technology-based therapies for PWD.
Lorenz et al. (2019)^*^	Mapping technologies for PWD and caregivers, classified by function, target user and disease progression.	Rapid Review, interviews and blog analysis. Forty-seven studies.	Online psycho-social support, cognitive training, psycho-education and remote monitoring of the PWD.	Most technologies target people with moderate and severe dementia living in their homes are focused on safety. Most technologies for PWD living in care homes are focused on care delivery and therapies. Memory aids and daily living technologies mostly target people with mild dementia living in their homes.	Little evidence back up the practical application of the identified technologies. Further researches should examine the impact of a wide range of technologies on the daily living.
Maia et al. (2018)	Analysis of interventions for PWD using ATs to sustain BADL.	Systematic Review, four studies.	Technology-based interventions to sustain BADL (safety, memory aid, monitoring, etc.).	Monitoring sensors has been evaluated as useful by PWD and caregivers; prompt systems facilitated medication adherence and finance management; navigation systems improved PWD's autonomy in movements. 1/4 study reported technical issues.	ATs are feasible to sustain PWD's BADL.
Neubauer et al. (2018)	Analysis of the types of technologies used to manage wandering behavior in PWD.	Scoping Review, 12 studies.	Interventions targeting PWD using sensors, alarms and locators to manage wandering.	26 types of technologies identified (GPS, sensors, alarms, Bluetooth, etc.). 67.7% of the devices were wearable. 7/12 studies reported positive results in managing wandering behaviors. The general acceptability was high.	Technologies can reduce risks associated with wandering behaviors and improve the autonomy in movements of PWD. Further studies are required to increase levels of evidence.
Pinto-Bruno et al. (2017)	Analysis of the validity and the efficacy of ICT-based interventions to promote social health and an active aging.	Systematic Review, six studies.	ICT-based interventions of reminiscence therapy, leisure activities, cognitive and physical training.	Qualitative – technologies foster social participation in PWD. Quantitative – People in the intervention group made more choices [t_(10)_ = 3.6717, *p* < 0.05] and sang more [t_(10)_ = 2.191, *p* < 0.05] than the control group. People in the control group spent more time asking questions [t_(10)_ = 3.13, *p* < 0.01] and initiated less conversations (*z* = 2.03, *p* < 0.05) than the intervention group.	Initial positive evidence emerged using ICT interventions. Specific outcomes measure to assess social health and social participation are needed for future studies.
Tyack and Camic (2017)	Analysis of the impact on well-being of touchscreen-based interventions for PWD.	Systematic Review, 16 studies.	Intervention using touchscreen devices to sustain reminiscence therapies, leisure activities, safety, communication and prompting systems.	Mixed results. Positive evidence on mood, involvement, perceived well-being and perceived satisfaction. A significant positive correlation emerged in one study between age and impact on mood (r_s_ = 0,46, *p* < 0.05), with greater effects on older people. Benefits of social interaction with relatives and on the sense of mastery (technological skills and satisfaction increasing).	Touchscreen-based interventions can improve the psychological well-being of PWD. More rigorous future studies are needed.
Yousaf et al. (2019)	Analysis of the evidence on the use of mHealth application for PWD.	Overview, 17 studies.	Interventions using mHealth Apps to sustain cognitive training, daily living, screening, safety, navigation and leisure activities.	Cognitive domain – available Apps target memory, communication, logical thinking, attention, language abilities and schedule. Screening domain – Apps target dementia detection and cognitive screening. Health/safety monitoring domain – Apps for fall detection and emergency help. Leisure domain – Apps for reminiscence therapy and socialization therapy. Navigation domain – Apps for tracking and location service.	Mobile health Apps are interactive, easy to use and independence promoting. These seems feasible AT intervention for PWD and caregivers.

* These studies are shown both in Table 5 and Table 7.

**Table 6:** New studies for PWD.

**References**	**Focus**	**Setting**	**Sample**	**Length**	**Methods**	**Interventions**	**Results**	**Conclusions**
Dethlefs et al. (2017)	Evaluation of the feasibility of computer-based cognitive stimulation using a spoken natural language interface.	Laboratory.	23 people, 13 healthy elderly, 10 PWD (mild to moderate).	20 min.	Pilot study non-RCT.	Computer-based cognitive stimulation (sorting, name recall, quiz and proverbs).	8/10 PWD enjoyed doing the activities. Correct answers and reaction time were similar between experimental and control groups. Quiz and proverbs activities were preferred over sorting and name recall.	It seems possible to convey cognitive stimulation through spoken natural language interface.
Favela et al. (2020)	Assess the benefits and limitations of using activity trackers for BPSD in dementia context.	Residential care facility.	10 PWD (mild to moderate dementia).	14 therapeutic sessions of 30 min.	Mixed methods design.	Cognitive stimulation therapy with the assistance of a social robot and activity trackers.	Activity tracker confirms or complements results obtained from the NPI-NH instrument or interviews with caregivers.	Activity trackers can help dementia research as they allow to gather data continuously and objectively.
Hung et al. (2018)	Feasibility and acceptability of an *iPad* intervention to support dementia.	Hospital.	Four PWD.	14 sessions of 15 min.	Mixed methods design.	Simulated presence therapy (1 min video pre-recorded by a relative).	Positive results in reducing BPSD and increasing mood and treatment adherence.	Simulated presence therapy using *iPad* can help PWD in hospital setting. Videos with a single person and a familiar voice are recommended.
Lancioni et al. (2020)	Smartphone-based intervention to manage goal-directed, walker-assisted ambulation and object use.	Residential care facility.	11 PWD (moderate dementia).	Multiple sessions of 3–5 min.	Mixed methods design.	Smartphone-based intervention to facilitate daily living.	Compared to a baseline of 0: - 2.9 correct target response of overall mean across participants; - over three indices of enjoyment/appreciation of overall mean across participants.	A smartphone-based intervention may be suitable to foster goal-directed, walker-assisted ambulation and object use.
Inel Manav and Simsek (2019)	Analysis of the effects of reminiscence therapy based on internet videos.	Residential care facility.	32 People with mild dementia.	60 min, once a week for 3 months.	RCT.	Reminiscence therapy using selected *YouTube* videos vs. traditional therapy.	Significate differences between experimental and control groups: SMMSE (Standardized Mini-Mental State Examination) (*p* < 0.01); ARS (Apathy Rating Scale) (*p* < 0.01).	Reminiscence therapy using internet-based videos improved the cognitive functions and apathy levels of people with mild dementia.
McCarron et al. (2019)	Analysis of the feasibility and utility of the Social Support Aid (*SSA*) App for PWD.	Domestic setting.	29 PWD, 19 MCI.	6 months.	Pilot RCT.	Intervention using the *SSA* App, combining a smartphone and a smartwatch, to help remembering names and relationships of familiar faces.	Utility – 3.10 (SD 0.63) pt. mean score using a 0–5 Likert scale with 15 items. No significant changes (*p* > 0.05) in quality of social interactions (PES-AD) or quality of life (DQoL). The App has been evaluated as too complex, stigmatizing and with an intricate enrollment process.	The App *SSA* did not improve the quality of life and the quality of social interactions of people with memory loss. More practical devices are required.
McGoldrick et al. (2019)	Analysis of the feasibility and utility of the memory aid App *MindMate*.	Domestic setting.	Three people with mild dementia.	5 weeks.	Three single cases.	Intervention using the App *MindMate* to sustain memory in daily life.	Significant memory improvement (*p* < 0.01) in participant A (from 49 to 93% correct tasks post training) and participant B (from 69 to 95%). Participant C withdrew from the intervention.	Use of *MindMate* seems feasible and effective in supporting memory in everyday tasks.
Moyle et al. (2019)	Analysis of the acceptability of telepresence robots in dementia care.	Laboratory.	Five PWD.	–	Mixed methods pilot study.	Videocall using the telepresence robot *Giraff*.	A sense of authenticity and social connection was experienced by participants. Significantly higher positive (mean score 18.77 ± 4.00) than negative affect (mean score 8.05 ± 1.76) on the I-PANAS-SF, and on the facial display subscale of the ODAS (positive – mean score 15.50 ± 3.51 vs. negative – mean score 4.00 ± 0.00).	Telepresence has potential use as it facilitates social connection in the dementia context. Further standardized studies are required to guide the implementation of telepresence in healthcare practice.
Obayashi et al. (2020)	Analysis of the impact of age, gender and the stage of dementia on an intervention using communication robots.	Residential care facility.	65 PWD.	8 weeks.	Non randomized quasi-experimental study.	Social assistance intervention using com-robots *COTA* and *PALRO*.	Participants aged ≥ 80 and in more advanced stage of dementia benefited more from the intervention (*P* < 0.05) than people younger and in less advanced stage. Selected items from the ICF (International Classification of Functioning, Disability and Health) scale were used.	The overall findings support the use of com-robots within the context of a care team for PWD.

**Table 7:** Interventions for caregivers.

**References**	**Focus**	**Methods**	**Interventions**	**Results**	**Conclusions**
Boots et al. (2014)	Analysis of the effectiveness, feasibility and quality of Internet interventions for informal caregivers of PWD.	Systematic Review. Twelve studies, three RCTs.	Informative websites providing information and strategies; peer-support/professional support through e-mails and phone calls.	Quantitative – small significant results in 6/12 on depressive symptoms, self-efficacy, perceived competence, decision-making and burden; Qualitative – positive outcomes on awareness, competence, mastery and perceived social support. Better outcomes for interventions combining informative websites with psycho-social support.	General positive outcomes emerged. Further researches are required due to small samples, not standardized designs and examined outcomes.
Brando et al. (2017)^*^	Analysis of the advantages and disadvantages associated with the implementation of technology into works with PWD and caregivers.	Literature Review (online-caregivers section of 3/30 studies).	Psycho-education online support through videoconference among peers.	Positive qualitative outcomes regarding online social support.	Few online interventions target caregivers. Initial evidence emerged.
Dam et al. (2016)	Analysis of interventions targeting caregivers focused on social support.	Systematic Review (remote support section of 15 studies, 11 RCTs).	Informative/psycho-education websites; peer-support through online forums and videoconferences.	Qualitative – Positive outcomes for the perceived social isolation, social support and the relationship with the PWD. Benefits for the decision-making process using informative websites; benefits for stress, depression, self-efficacy and burden using videoconferences.	Positive qualitative outcomes emerged. Future research should use more standardized designs.
Egan et al. (2018)	Analysis of online interventions targeting caregivers of PWD.	Systematic Review. Eight RCTs.	Psycho-education, psychotherapy (cognitive reframing and relaxation) and cognitive training using forums, videoconferences and selected videos.	Positive evidence on depressive symptoms 2/8, anxious symptoms 2/8, acquired skills 2/8 and self-efficacy.	Positive evidence emerged from heterogeneous designs; more standardized studies are required. No benefits emerged on the QoL.
Godwin et al. (2013)	Analysis of the effectiveness of interventions targeting caregivers of PWD.	Systematic Review. Eight studies, four RCTs.	Informative websites; psycho-social support through forums, e-mails, chats and videos.	Positive benefits on depressive symptoms 4/8 and anxious symptoms 2/8. Mixed evidence on the social support.	Mixed results and heterogenous designs were found. Future standardized RCTs are required.
Hopwood et al. (2018)	Analysis of the effectiveness of online interventions targeting caregivers of PWD.	Systematic Review. Forty studies, nine RCTs.	Psycho-social support in group of peers, using chats, forums, videoconferences and avatar 3D; training on the decision-making process.	Benefits on depressive and anxious symptoms; increased self-efficacy and QoL. Peer-support is more appreciated if conducted through videoconferences; informative interventions are preferred via websites rather than using handbooks.	Mixed results, but mostly positive, emerged. Future studies should better examine the caregivers' specific needs.
Jackson et al. (2016)	Analysis of telephone-based and internet-based interventions targeting caregivers.	Systematic Review. Twenty-two studies, five internet-based.	Psycho-education, psychotherapy and psycho-social supportive interventions.	Qualitative positive results on depressive symptoms, general mental health and QoL.	Interventions combining different programs and devices had led to better outcomes.
Leng et al. (2020)	Analysis of the efficacy of internet interventions in improving health of caregivers of PWD.	Systematic Review and Meta-Analysis. Seventeen RCTs.	Online psycho-social and psycho-educative support.	Significant positive results on depressive symptoms (SMD = −0.21; 95% CI −0.31 to −0.10; *P* < 0.001), perceived stress (SMD = −0.40; 95% CI−0.55 to −0.24; *P* < 0.001), anxious symptoms (SMD = −0.33; 95% CI−0.51 to −0.16; *P* < 0.001) and self-efficacy (SMD = 0.19; 95% CI 0.05–0.33; *P* = 0.007). No significant improvements were found in caregiver burden, coping, caregiver's reactions to behavioral symptoms and quality of life.	Internet-based interventions are generally effective at improving the health of family caregivers.
Lorenz et al. (2019)^*^	Mapping technologies for PWD and caregivers, classified by function, target user and disease progression.	Rapid Review, interviews and blog analysis.	Online psycho-social support, cognitive training, psycho-education and remote monitoring of the PWD.	Most technologies targeting carers are focused on memory sustainment and care delivery.	Little evidence back up the practical application of the identified technologies. Further researches should examine the impact of a wide range of technologies on daily living.
Lucero et al. (2019)	Analysis of the effectiveness on health of ICT-based interventions targeting caregivers.	Systematic Review. Twelve RCTs, 6 internet-based.	Online psychotherapy; informative and educative interventions using websites; cognitive and physical training.	Internet interventions -> increased positive affect (*P* = 0.01), decreased concerns for PWD's behaviors (*P* = 0.02). Internet + telephone -> increased satisfaction (*P* = 0.015), physical activity (*P* < 0.01), self-efficacy (*P* < 0.01); decreased depressive symptoms (*P* = 0.02), anxious symptoms (*P* = 0.01) and stress (*P* < 0.05).	Positive benefits on health emerged from heterogeneous studies. A standardized methodology is required.
McKechnie et al. (2014)	Analysis of computer-based interventions targeting caregivers of PWD.	Systematic Review. Twelve studies, six RCTs.	Psycho-education, psycho-social support among peers, psycho-social support with health care professionals.	Positive significant results (*P* < 0.05) on: depressive symptoms (four studies), general mental health (three studies), burden and stress (five studies), social support (one study), positive aspect of caregiving (one study) and self-efficacy (two studies).	Depression and burden were the most examined outcomes. Future studies should use a standardized methodology and examine the same outcomes.
Parra-Vidales et al. (2017)	Analysis of online psycho-educational interventions targeting caregivers of PWD.	Systematic Review. Seven studies.	Online informative support, psycho-social support among peers and with health care professionals through chat and videoconference; cognitive training.	Positive results on self-efficacy (two studies), acquired knowledge (two studies), functional autonomy (one study), anxious/depressive symptoms (one study).	Outcomes examination is often not reported or not standardized. Online interventions have benefits on the social aspect and are usually perceived as positive.
Rathnayake et al. (2019)	Analysis of interventions based on mHealth-App targeting caregivers.	Integrative Review. Seven studies.	Information, psycho-education, cognitive training and PWD's monitoring.	The main focus areas of mHealth App studies are categorized as: carer education, monitoring and cognitive training.	A theoretical model is required to guide the designing of mHealth App interventions. Further researches should focus more on psycho-education and psycho-social support.
Ruggiano et al. (2018)	Analysis of technology-based interventions targeting caregivers of PWD living in rural areas.	Systematic Review. Thirty studies, 18 RCTs.	Online interventions focused on psycho-social support groups, psycho-education, psychotherapy, monitoring of mental and physical health.	Significant positive benefits (*P* < 0.05) on: depressive symptoms (five studies), anxious symptoms (two studies), other psycho-social aspect (six studies), as stress and perceived support. No benefits on self-efficacy and caregiver's skills emerged.	Few studies identified their sample population as living in rural areas. Future interventions should analyze the needs of the specific population.
Scott et al. (2016)	Analysis of the effectiveness of TB-CBT interventions targeting caregivers of PWD.	Systematic Review. Four studies.	Cognitive-behavioral therapy using the internet and selected multimedia.	Small significant effects post-intervention of the TB-CBT on depression (*P* = 0.04); equivalent to the traditional CBT.	Future studies should analyze long-term effects of the TB-CBT. TB-CBT is an economical alternative to the traditional CBT.
Waller et al. (2017)	Analysis of the acceptability, utilization and effectiveness of ICT-based interventions targeting caregivers.	Systematic Review. Online section of 19 studies.	Online psycho-education, cognitive training, psycho-social support among peers and with health care professionals.	Heterogeneous positive evidence emerged on general mental health, depressive symptoms, burden, positive aspect of caregiving and perceived social support. A positive acceptability emerged.	Potential benefits of ICT-based interventions emerged. High-quality studies are required to detect the most recommended types of intervention.

* *These studies are shown both in Table 5 and Table 7*.

## Results

Results are sorted into two main sections, one per target population involved. As shown in Table 3, 12 out of the 30 reviews are related to online interventions targeting caregivers, 16 of them concern PWD, and two reviews target both PWD and caregivers. On the other hand, all of the nine new studies address PWD issues.

### Quality Assessment of the Included Reviews

The quality assessments regarded the extent to which the 23 systematic reviews and seven reviews met the inclusion criteria.

All the systematic reviews received an AMSTAR score between 5 and 9, with a mean score of 7.4 (standard deviation = 1.2). All reviews were designed a priori (AMSTAR item 1); more than half of the analyzed works indicated that study selection and data extraction were performed by two authors minimum (item 2); all the reviews were based on electronic searches (item 3); 14 reviews included the status as an analyzed criterium (item 4); no reviews provided a list of excluded studies (item 5); all the reviews but one provided tables displaying the characteristics of the analyzed studies (item 6); 17 out of 23 reviews performed the study quality assessment (item 7); all the reviews but one based their conclusion on study quality levels (item 8); only four reviews performed a meta-analysis (item 9); 10 reviews reported publication bias (item 10), and 21 reviews discussed the conflicts of interest (item 11).

The reviews not analyzed via AMSTAR scores were subjected to the SANRA process (Brando et al., 2017; Dove and Astell, 2017; Klimova and Maresova, 2017; Neubauer et al., 2018; Lorenz et al., 2019; Rathnayake et al., 2019; Yousaf et al., 2019). Overall, the studies achieved a score of 11.1, with a standard deviation of 0.8. Across the items (i.e., justification of the article's importance for the readership, statement of concrete aims or formulation of questions, description of the literature search, referencing, scientific reasoning, and appropriate presentation of data), and no study scored 0.

**Figure 2:**
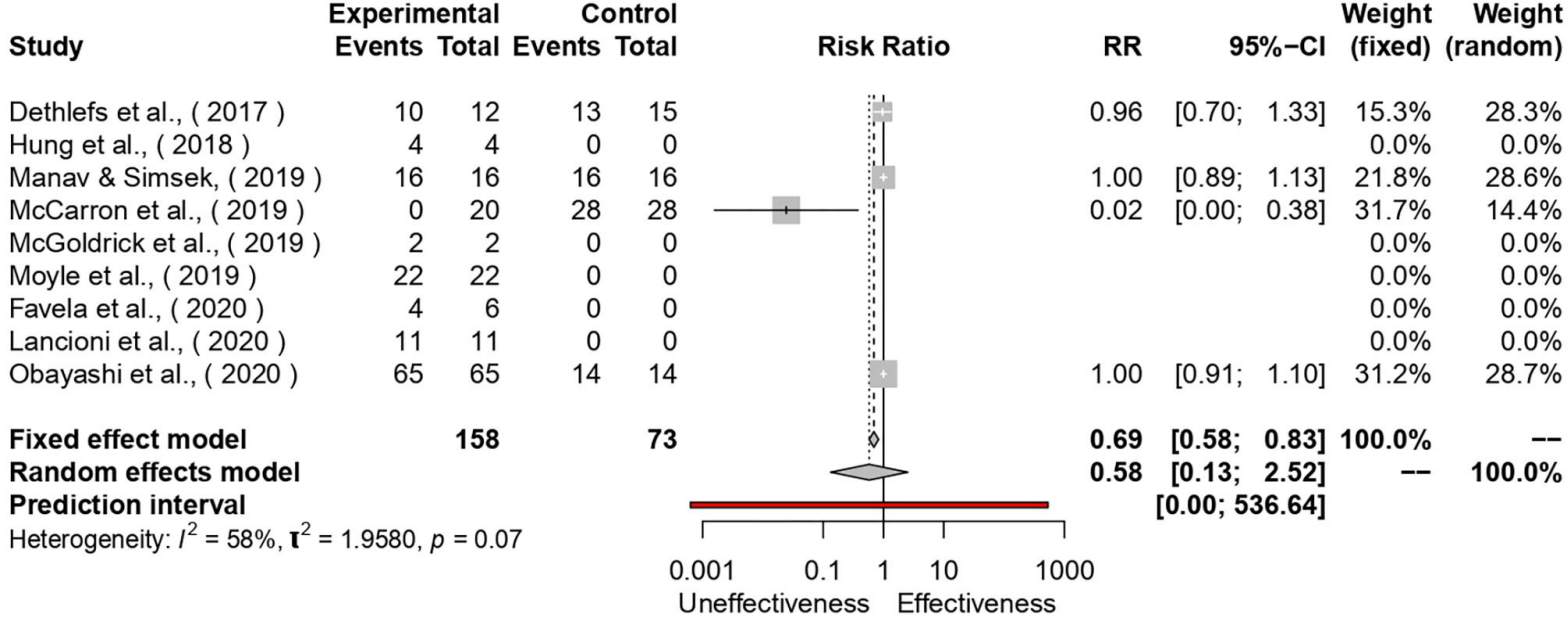
The figure displays new studies' effect size.

Finally, we performed a risk ratio and a forest tree calculation to understand the effectiveness of the new studies. If the risk ratio was calculated higher than one, the study's technologies have an effective impact on the target (Figure 2).

### Interventions for PWD

We classify the interventions targeting people with dementia into the four following macro-categories. Monitoring and security included studies with remote technologies aimed at detecting risky behavior and compensating for environmental obstacles. Daily living sustainment contains studies investigating devices supporting the PWD's cognitive functions. The therapeutic technology-based interventions were split between the studies that investigated cognitive aspects and the ones addressing psychosocial care.

#### Monitoring and Security

The interventions regarding monitoring and security appear to be analyzed in 10 reviews and one new study from our analysis. Lorenz et al. (2019) underline how technology targeting PWD living in their homes is mainly aimed at monitoring them or increasing the environment's security. The most used devices for achieving this purpose are video cameras. These devices might be used to ascertain the person in real time or record tapes for later analysis (Lorenz et al., 2019; Yousaf et al., 2019). The included reviews also reveal how video monitoring allows us to increase medication compliance in people with dementia (Fleming and Sum, 2014; El-Saifi et al., 2018). Video monitoring also leads to relevant benefits inside care homes, and it happens when it is combined with bed sensors. Both these technologies allow us to reduce intrusive check-ups overnight from healthcare professionals by avoiding sudden and unnecessary awakenings. Despite a few technical issues and the false alarms that emerged during the studies, the devices lead to a positive quality of life-related outcomes and high levels of acceptance from either PWD, their caregivers, and staff members (Maia et al., 2018; Daly Lynn et al., 2019). Finally, one review suggests the adoption of the actigraphy technique as having potentially worth in helping to monitor people in care home settings (Favela et al., 2020).

Other relevant devices for monitoring are the position trackers and locators. These are usually based on GPS technology featuring most of the ordinary smartphones. Neubauer et al. (2018) analyzed monitoring technologies for PWD and reported that GPS devices are usually implemented in wearable items, such as belts or wristwatches. *CANDEROID*, for example, is a system based on a wrist sensor combined with a smartphone App allowing the caregiver to monitor and track the position of the PWD in real-time (Brando et al., 2017). Benefits to the perceived security and quality of life emerge from using these technologies by PWD and informal caregivers (Lorenz et al., 2019). Moreover, as some of the devices imply the active roles of PWD, they and their caregivers can contact each other to ask for mutual information or help (Lorenz et al., 2019).

Aside from the beneficial impact these devices offer, some technical issues can emerge in GPS-based technologies, such as position inaccuracy or signal instability (Fleming and Sum, 2014). However, due to the technological progression, these problems have been fixed insomuch as they become a useful aid in managing wandering behaviors (Neubauer et al., 2018; Lorenz et al., 2019).

Among security systems, many studies focus on smart-homes technology aimed at reducing risk and increasing the quality of life of the home denizens (Fleming and Sum, 2014; Tyack and Camic, 2017; Neubauer et al., 2018; Brims and Oliver, 2019; Daly Lynn et al., 2019; Lorenz et al., 2019). In domestic settings, automatic sensors are used to detect sudden heat changes, gas leakages, forced doors opening, and so on or to facilitate the management of light switches and water valves (Lorenz et al., 2019). An example is the *COGNOW* program, which capitalizes on a central control panel capable of administrating all the different technological tools implemented in the house (Tyack and Camic, 2017).

Another relevant topic is related to the use of technology for fall prevention. Positive outcomes are observed both with basic support, such as light pathways on the ground or bright handrails, and with a more complex system, such as electronic armbands combined with modern sensors, which send alarms to an assistance center in case of an emergency. Specifically, three randomized controlled studies show that the fall risk featuring the experimental groups is 50% lower than those expressed in the control groups. Moreover, it emerges that the use of ATs decreases the number of risky behaviors, as leaving the home incautiously might result in negative consequences. In contrast, for what concerns the quality of life and the reduced institutionalization, no significant positive outcomes emerged from these studies (Brims and Oliver, 2019).

In care home settings, tagging systems can be implemented: they can be envisioned as intangible spatial barriers that PWD should not overstep during specifically scheduled times; otherwise, an alarm would start ringing (Fleming and Sum, 2014). Tagging technologies are well-accepted both by PWD and healthcare professionals, as they are less obtrusive than physical constraints (Neubauer et al., 2018). Moreover, tagging systems increase both the perceived and the actual safety (Daly Lynn et al., 2019). In contrast, the devices that limit people's autonomy, such as electronic lock doors, are not well-accepted, as they are perceived as dehumanizing despite the improved safety of PWD (Neubauer et al., 2018). In terms of psychological outcomes, non-constraining technologies show positive benefits on the levels of PWD's perceived well-being and anxiety (Neubauer et al., 2018). Lastly, the reviews show that the alarms placed between the rooms are associated with positive qualitative outcomes in care home settings (Yousaf et al., 2019).

#### Daily Living Sustainment

The issues of interventions regarding daily living sustainment using ATs appear in seven reviews and four new studies from our analysis. Daily living sustainment is the primary purpose of ATs with PWD (Lorenz et al., 2019). Indeed, ATs can support cognitive functions, such as different memory types, spatio-temporal orientation, and language. For what concerns the prospective memory, devices like digital organizers and electronic reminders can improve the quality of life of PWD (Brando et al., 2017; Lorenz et al., 2019; Lancioni et al., 2020). An example is the App *MindMate*, an electronic calendar designed to help PWD remember the daily schedule. The App has been evaluated arranging simple tasks, such as “call the researcher,” which people with dementia had to pursue at scheduled times. After 5 weeks of intervention, *MindMate* showed benefits on the prospective memory of the PWD (McGoldrick et al., 2019). Other devices that are used to sustain prospective memories are pill dispensers. Controversial outcomes are associated with these technologies: some authors describe them as functional (Fleming and Sum, 2014; Maia et al., 2018), and others suggest that their usage is too complicated for PWD (Daly Lynn et al., 2019).

Regarding procedural memory, instead, positive outcomes emerge from using prompting systems. These consist of tools giving step-by-step prompts, either in visual or vocal forms, to guide PWD to achieve daily tasks as cooking, washing their hands, and setting the table correctly (Brando et al., 2017; Maia et al., 2018; Daly Lynn et al., 2019). Moreover, some potential benefits emerge from using mobile Apps to guide practical actions (Lancioni et al., 2020).

There is little evidence to endorse the application of ATs to sustain the memory of faces. The only identified intervention in this area concerns a randomized controlled trial (RCT) using an App called *SSA* (“*Social Support Aid*”). The App combines a smartwatch camera with an online database containing preloaded faces labeled with a personalized tag, such as “Emma, daughter.” The system aims to match the faces included in the database and the ones caught by the smartwatch camera. Once the match has finished, it notifies the person with the assigned tag in case of a positive match. PWD evaluates the App as too complicated, and it does not increase users' quality of life (McCarron et al., 2019).

For what concerns the spatio-temporal orientation, ATs are useful to sustain daily living. Apart from the already described electronic calendars, other devices are employed with PWD, such as monitors capable of harmonizing night-time awakenings in care home settings. These devices are usually placed in front of the PWD's bed, showing recommendations like “it's night, let's go back to sleep” and similar (Lorenz et al., 2019; Moyle et al., 2019). Moreover, it emerges that robot-assisted navigation can lead to positive outcomes to increase PWD's ability to move autonomously (Maia et al., 2018).

Regarding language, positive outcomes derive mainly from using smartphones in functional manners. In particular, improvements in the semantic component of language emerge when PWD use their smartphones to take notes of words or navigate the internet when they do not remember a definition (Brando et al., 2017). In the same fashion, Lorenz et al. (2019) report positive daily PWD experiences using *SIRI*, a famous virtual assistant. More evidence emerges regarding technologies to support the communication process: PWD answers more frequently to the incoming calls when the telephones are adapted to their perceptual and cognitive needs. Differently, these devices cannot improve the quality of life of the users ultimately, as they cannot solve broader problems such as remembering to call or whom the PWD have spoken to (Topo et al., 2002; Fleming and Sum, 2014).

Another piece of evidence supports the employment of telepresence robots to sustain social connections. It emerged from the pilot study led by Moyle et al. (2019) using the com-robot *Giraff* in an experimental setting with five PWD. *Giraff* is a wheel-based, remotely controlled device carrying a tablet that can convey videocalls. *Giraff* is also of human height, and the upper part of the robot may also be bent forward or tilted left and right, simulating social head gestures. The devices were evaluated as realistic and useful by four out of five PWD. Furthermore, the most appreciated aspect was the possibility to control Giraff remotely to make it move around the room.

Moreover, even some social aspects can be sustained by smart-home devices. Systems like the *PAL4-Dementia* allow both to manage the technological tools implemented in the house and start video calls with relatives or healthcare professionals (Tyack and Camic, 2017).

Although we observe that ATs interventions usually lead to positive outcomes for what concerns daily living, there is also a reported discrepancy between the experimental research and the actual uptake of the devices in everyday life. In fact, in this regard, some authors noted that the use of technology decreases in the follow-up because of disease development, limited financial resources, time- and burden-related constraints (Fleming and Sum, 2014; Lorenz et al., 2019).

#### Cognitive-Focused Therapeutic Interventions

From our analysis, the interventions regarding therapeutic support emerge in 13 reviews and three new studies. Technological devices, Apps, videoconferences, and software, convey and support different intervention categories targeting the PWD's cognitive functions, i.e., cognitive training, stimulation, and rehabilitation. García-Casal et al. (2017) carried out a review and meta-analysis of 12 computer-based cognitive intervention studies. Once they have analyzed the outcoming effects regardless of the intervention category, they report intermediate results regarding cognition and anxiety and small impacts concerning depression. In contrast, no consequences were observed in terms of the PWD's daily activities.

For what concerns cognitive training, mixed outcomes emerge among the analyzed reviews targeting people with dementia. In a review of four RCTs, Klimova and Maresova (2017) reported that half of the studies do not produce significant outcomes. One of them reported benefits on episodic memory and abstract reasoning, while the remaining study highlighted that computer-based cognitive interventions can slow down cognitive decline. Moreover, it emerges that the *mHealth* App can train, monitor, and self-assess the performance achieved in all the cognitive functions. Indeed, the Apps help PWD function better in their daily lives, especially if the users accept it because it is intuitive (Yousaf et al., 2019).

Technological devices mainly support cognitive stimulation interventions since stimulation is the most appreciated activity by PWD (Liapis and Harding, 2017; Daly Lynn et al., 2019), and it leads to an extensive generalization of the benefits (Brando et al., 2017).

Our analysis suggests that different stages of the dementia pathway are associated with preferred stimulation activities. People with mild or moderate dementia tend to prefer challenging tasks, such as the ones provided via videogames modality. Among videogames, an example is the software *Big Brain Academy*, which has a positive impact on perception, memory, logical reasoning, and general cognitive functioning (Brando et al., 2017). On the other hand, people with severe dementia prefer more static and sense-based activities, such as listening to music or watching videos (Liapis and Harding, 2017).

Besides, implementing a technological component in stimulation interventions allows us to compensate for sensory deficits thanks to apposite designed interfaces. For example, headphones and image projectors boost the auditory and the visual apparatus, respectively (Lazar et al., 2014). Additionally, many benefits emerge using innovative input systems, such as touchscreens, motion-sensors, and voice user interfaces. Tyack and Camic (2017) report that the interventions based on intuitive touchscreens led to positive mental health outcomes, perceived well-being, and satisfaction, especially in older people. Moreover, the request of learning how to use modern devices increases PWD's involvement, pride, and sense of mastery (Tyack and Camic, 2017). Tablets are also frequent in these programs of intervention. By reproducing multimedia or allowing PWD to express their art capacity, tablets support behavioral symptoms management while sustaining people's creative skills. The App *ExPress Play*, for example, can generate chord-based music thanks to the touchscreen (Tyack and Camic, 2017; Yousaf et al., 2019). In a similar vein, while studying the software recognizing and synthesizing human voices, Dethlefs et al. (2017) report that PWD appreciate voice user interfaces, as can be seen by increased involvement in computer-based cognitive stimulation programs. Again, devices based on motion sensors are highly recommended for PWD because they can compensate for the issues arising from memorizing device button-keys (Dove and Astell, 2017). Moreover, benefits emerge regarding the PWD's cognitive decrease associated with the disease, while enhancing their moods positively, as they stimulate people's movements (Dove and Astell, 2017).

For what concerns cognitive rehabilitation, instead, the literature shows that virtual reality can be a component used to recreate settings that are familiar to PWD or to let them practice with the execution of daily activities, such as cooking or shopping at the grocery shop. The adoption of the virtual setting produces better outcomes on the general cognitive functioning, learned skills, self-efficacy, and motivation, with respect to practicing the same activities within traditional environments. Moreover, by combining virtual reality headset and controller, visuospatial orientation and autonomous movements can increase (Brando et al., 2017).

Finally, some evidence related to therapeutic interventions remotely conducted using videoconferences emerge from the literature search. It is revealed that online memory clinics are positively accepted by PWD and mostly by living in rural areas (Weiner et al., 2011; Lorenz et al., 2019). Also, two RCT studies highlight that remote cognitive interventions produce benefits on PWD's general cognitive functioning, attention, memory, calculus, and phonemic and semantic verbal expression (Jelcic et al., 2014). Furthermore, it is revealed that some PWD and their caregivers express specific preferences for remotely conducted interventions since they limit laborious transfers (Fleming and Sum, 2014).

#### Psychosocial Interventions for PWD

From our analysis, we yield that technological devices can support psychosocial interventions such as reminiscence, light therapy, multisensory therapy, simulated presence therapy, and therapy based on social robots. Lorenz et al. (2019) point out that technologies can easily convey psychosocial interventions in home-care settings. For what concerns reminiscence therapy, technological devices might be useful as they allow you to select personalized multimedia and adequately stimulate the emotional memory (Inel Manav and Simsek, 2019). For example, in an RCT focused on the effects of the reminiscence therapy, *YouTube* videos obtain positive results that concern both PWD's cognition and mood (Inel Manav and Simsek, 2019). Moreover, by integrating a camera in smart-watches devices, it becomes possible to gather pictures or videos during daily living. The collected multimedia becomes useful vehicles during reminiscence therapy sessions (Lazar et al., 2014). Again, as soon as reminiscence therapy meets technological devices, such as with touchscreen interfaces, people's involvement increases. Indeed, people can autonomously feel competent and capable of handling the digital contents (Liapis and Harding, 2017; Tyack and Camic, 2017; Yousaf et al., 2019). Besides, the increased confidence in modern devices represents an opportunity to close the gap between social generations (Yousaf et al., 2019). The “*Computer interactive reminiscence and conversation aid – CIRCA*,” i.e., a program targeting the dyad PWD-caregiver, has led to positive results in the decision-making process and the social involvement and in particular for singing activities (Pinto-Bruno et al., 2017; Tyack and Camic, 2017).

Finally, technology allows to overcome environmental barriers and to conduct therapies from a remote position because they provide the opportunity to both communicate and access the same multimedia simultaneously (Lazar et al., 2014; Dethlefs et al., 2017). Dyads positively accept remotely delivered therapies because they can ameliorate the management of behavioral symptoms (agitation, irritability, and insomnia) (Lazar et al., 2014). In particular, *MyBrainBook* is an online platform aimed at conveying reminiscence therapy. By connecting PWD and their relatives and friends, they can still feel part of a social network. Moreover, as it capitalizes on a cloud environment to gather personalized content, it is useful when implementing the process of reminiscence (Dethlefs et al., 2017). Positive results also came out from interventions using tools to start the required applications for the therapies remotely. Such a strategy allows us to compensate for the lack of technological skills featuring some people (Yasuda et al., 2013; Lazar et al., 2014).

As it emerges for the reminiscence, even light therapies can be aimed at managing behavioral symptoms and they are mainly conducted in home-care settings. In particular, positive effects followed in the forms of agitation, circadian rhythms, and well-being (Fleming and Sum, 2014; Daly Lynn et al., 2019). Similar benefits come out using multisensory therapies, especially with the Snoezelen Room, which leads to positive results regarding well-being, and behavioral agitation. Despite this evidence, multisensory therapies have emerged to produce fewer effects than the immersion in real natural environments (Fleming and Sum, 2014).

Simulated presence therapies capitalize on technological devices; they involve videos that were pre-recorded by a family member. The videos recorded using spontaneous language lead to well-being improvement, fewer phone-calls during the night-time, and increased adherence to medical recommendations and compliance (Fleming and Sum, 2014; Hung et al., 2018; Daly Lynn et al., 2019).

The therapies based on social robots appear to produce contrasting evidence (Daly Lynn et al., 2019). Fleming and Sum (2014) highlight that adding a mechanical component does not lead to better improvements than therapies using regular pet plushies. Other authors, instead, report positive benefits on behavioral agitation, depressive symptoms, and social interactions, using the pet robots *PARO, NeCoRo, AIBO*, and *CuDDler* (Daly Lynn et al., 2019). On the other hand, using the communication robots *COTA* and *PALRO*, positive results emerge even with regards to the functional autonomy of PWD, especially for people over 80 with severe dementia (Obayashi et al., 2020).

### New Studies Effectiveness

A total of six studies out of 10 did not use a control group. All of them but McCarron et al. (2019) reported the positive effects of technologies. The heterogeneity of the study pool was almost significant (See Figure 2). Once all the studies without control groups, with the addition of McCarron et al., were removed, the remaining three works showed a homogeneous risk ratio [RR = 1, 95%- CI: 0.93; 1.08]. As described previously, the study of McCarron et al. showed that no positive social engagement emerged in the 20 people enrolled in the smart-watch use compared to the 28 counterparts.

### Interventions for Caregivers

The interventions targeting caregivers capitalize on different web interfaces and services. In particular, Hopwood et al. (2018) highlight how online interventions might be delivered either via private or public services. Private services include online tools available only for a restricted number of people, with access granted upfront invitation and/or after registration. These systems allow for the exchange of personal information, ensuring privacy protection. From our analysis, e-mails (Godwin et al., 2013; Boots et al., 2014; McKechnie et al., 2014; Dam et al., 2016; Hopwood et al., 2018), chats (Boots et al., 2014; McKechnie et al., 2014; Dam et al., 2016; Parra-Vidales et al., 2017; Waller et al., 2017; Hopwood et al., 2018) and videoconferences (Boots et al., 2014; McKechnie et al., 2014; Dam et al., 2016; Scott et al., 2016; Brando et al., 2017; Parra-Vidales et al., 2017; Waller et al., 2017; Egan et al., 2018; Hopwood et al., 2018; Ruggiano et al., 2018; Lorenz et al., 2019) emerge to feature the interventions for caregivers. Moreover, the evidence applying an online virtual setting with 3D avatars was useful to help caregivers communicate with each other while preserving a sense of privacy (O'Connor et al., 2014; Hopwood et al., 2018).

Public services include free-access content at everyone's disposal, such as the frequently used informative websites (Godwin et al., 2013; Boots et al., 2014; McKechnie et al., 2014; Brando et al., 2017; Parra-Vidales et al., 2017; Hopwood et al., 2018; Ruggiano et al., 2018; Lucero et al., 2019). Moreover, other services, such as blogs (Hopwood et al., 2018), forums, or selected social networks (Godwin et al., 2013; Boots et al., 2014; McKechnie et al., 2014; Dam et al., 2016; Parra-Vidales et al., 2017; Egan et al., 2018; Hopwood et al., 2018; Lorenz et al., 2019) and multimedia (Boots et al., 2014; McKechnie et al., 2014; Jackson et al., 2016; Scott et al., 2016; Brando et al., 2017; Egan et al., 2018; Hopwood et al., 2018; Ruggiano et al., 2018; Lucero et al., 2019) might be either private or public, as a function of the privacy settings set by the admin. Finally, it emerges that *mHealth* Apps for smartphones or tablets are used in online-based interventions for caregivers of PWD (Brando et al., 2017; Rathnayake et al., 2019).

Regarding the aims featuring the AT-based interventions, the analysis of the reviews suggests that we group the intervention aims into six groups: informative, psycho-education programs, psychosocial support, psychotherapy, cognitive training, and physical training. Informative interventions are deployed through websites providing information on many issues, such as the treatment and the management of dementia, the risks associated with the disease, and the implication on caregivers' health. Moreover, they provide useful links and contact information for community services (Godwin et al., 2013; Rathnayake et al., 2019) and are usually part of multicomponent programs (Boots et al., 2014; Brando et al., 2017; Hopwood et al., 2018; Lorenz et al., 2019).

Psycho-education programs mainly target caregivers' strategies and coping skills. Private services are preferred over public ones. When people seek help from healthcare professionals, such assistance can arrive through videoconferences or by watching recommended and personalized educative videos (Jackson et al., 2016; Parra-Vidales et al., 2017; Waller et al., 2017). Also, the *mHealth* App attempt was for the same purpose (Rathnayake et al., 2019). As it happens for informative interventions, even the psycho-education ones are often part of multicomponent programs together with psychosocial support or psycho-therapeutic interventions (Boots et al., 2014; McKechnie et al., 2014; Scott et al., 2016; Brando et al., 2017; Egan et al., 2018; Ruggiano et al., 2018; Lorenz et al., 2019; Lucero et al., 2019).

Psychosocial supportive interventions are focused on the improvement of caregivers' emotional well-being and social health through videoconferences among small groups of peers, chats, e-mails, or self-administered personalized multimedia content (Boots et al., 2014; McKechnie et al., 2014; Dam et al., 2016; Jackson et al., 2016; Waller et al., 2017; Egan et al., 2018; Hopwood et al., 2018; Ruggiano et al., 2018; Lorenz et al., 2019; Lucero et al., 2019). The participation of a healthcare professional is not mandatory (Hopwood et al., 2018).

Psycho-therapeutic interventions usually aim to reduce depressive or anxious symptoms and dealing with caregivers' burdens. The cognitive-behavioral approach is popular; meanwhile, cognitive reframing and relaxation are the most frequently applied techniques. For these interventions, the preference for the videoconferences has overcome the one for written communication (Boots et al., 2014; McKechnie et al., 2014; Jackson et al., 2016; Brando et al., 2017; Egan et al., 2018; Lorenz et al., 2019). Besides, the monitoring of the caregiver's emotional state is an essential aspect of the process: through the *mHealth* App, well-being-related symptoms can be self-assessed and shared with care providers together with other medical records (Brando et al., 2017; Rathnayake et al., 2019).

Interventions for caregivers based on cognitive and physical training promote healthy lifestyles and future healthy aging. The cognitive practice usually targets decision-making and problem-solving processes (Boots et al., 2014; Waller et al., 2017; Egan et al., 2018; Ruggiano et al., 2018; Lorenz et al., 2019). On the other hand, physical training pertains to easy motor exercises (Ottoboni et al., 2018; Ruggiano et al., 2018; Lorenz et al., 2019; Lucero et al., 2019). Both types of training are delivered to small groups of users via videoconferences with healthcare professionals or via written chats or forums (Hopwood et al., 2018). Finally, cognitive function and physical health might be self-assessed using specific mobile Apps (Brando et al., 2017; Rathnayake et al., 2019).

Overall, the literature suggests the need to match aims, interventions, and interfaces. Once the purposes are defined through needs and capacity assessment, interventions obtain better results if they fit with the appropriate interfaces (Ajzen, 1985). Informative websites are preferred over handbook instructions and seem to be the best way to provide fast and straightforward resources (Hopwood et al., 2018). Differently, videoconferences are the preferable interventions to improve caregivers' emotional well-being and to communicate in small groups of peers either in public forums or through private messaging (Dam et al., 2016; Parra-Vidales et al., 2017; Waller et al., 2017; Hopwood et al., 2018). Moreover, peer support seems to entail the best way to improve decision-making processes and increase caregivers' confidence in their choices. Finally, it appears usually more appreciated when it integrates multicomponent programs (Godwin et al., 2013; Hopwood et al., 2018).

As seminally suggested elsewhere (Moniz-Cook and Manthorpe, 2009), even here, the interventions that are capable of combining different modalities lead to better outcomes. The combination of videoconferences with phone calls and/or informative websites produces higher positive effects than those obtained using a singular channel. In particular, positive outcomes emerged related to emotional well-being, self-efficacy and perceived satisfaction, and self-efficacy and perceived satisfaction (Boots et al., 2014; Jackson et al., 2016; Lucero et al., 2019). Indeed, in general, interventions provided positive results. The main benefits regard emotional well-being (depression, anxiety, stress, and burden), learned skills (decision making, knowledge, self-efficacy, and strategies), and social aspects (perceived support and positive aspects related to caregiving, such as bonding with your relative; (McKechnie et al., 2014; Dam et al., 2016; Egan et al., 2018; Ruggiano et al., 2018). Moreover, despite the few quantitative analyses and the limits concerning the adopted methodologies, results highlighted the benefits online interventions have for what concerns caregivers' quality of life (Boots et al., 2014; Waller et al., 2017; Leng et al., 2020).

## Discussion and Conclusion

The present review analyzes the role of technology in the interventions addressed toward both PWD and their caregivers. The final summary aims to provide tangible support to decision-makers in deciding which ATs may better compensate for the dysfunctionalities featuring many dementia contexts.

The quality of the analyzed literature was high. Both the AMSTAR and the SANRA scores returned adequate standard levels, notwithstanding the reported methodologies' heterogeneous quality.

From our analysis, it emerges that in dementia contexts, the use of ATs is increasing. Such technologies can facilitate daily living, either for what concerns daily activity and the possibility to connect people that are geographically distant. Connections are particularly relevant in the case of difficulties associated with psychological states, personal injuries, and orographic features. In all these cases, technology can compensate for the limitations imposed on traditional human interactions. It represents a useful resource to stay in touch with relatives, friends, and physicians or therapists, too (Novitzky et al., 2015; Cheung and Peri, 2020).

In this light, ATs can become useful even to face social distancing occurring during further pandemic waves. Monitoring technologies, such as video-cameras or GPS-based systems, meet the visit restrictions and thus contagion by reducing the number of check-ups both in residential settings and PWD's homes (Fleming and Sum, 2014; Brando et al., 2017; Tyack and Camic, 2017; Neubauer et al., 2018; Lorenz et al., 2019). Simultaneously, ATs can compensate for the distress associated with the resulting isolation through communication tools designed to keep people remotely “in-touch.” Phone-calls, chat interfaces, videoconferences, and remote therapies, for example, can connect family members, physicians/therapists, and communities of peers (Weiner et al., 2011; Jelcic et al., 2014; Dethlefs et al., 2017; Lorenz et al., 2019; Cheung and Peri, 2020). Moreover, telepresence robots may be useful surrogates during isolation by increasing daily stimulation activities. In the same vein, even multimedia, Apps offering interactive gaming or automatic prompting systems can either stimulate cognitive functions or sustain PWD daily living and instrumental activities (Brando et al., 2017; Tyack and Camic, 2017; Daly Lynn et al., 2019; Moyle et al., 2019). However, people with dementia are not the only ones who can take advantage of different technological tools. Remote ATs can involve PWD's caregivers by providing them with several types of supportive programs, which, in turn, emerged to have many positive outcomes. If, on the one hand, the number of online or remote supportive tools targeting PWD are few, they are positively evaluated both by PWD and their caregivers (Weiner et al., 2011; Lazar et al., 2014; Lorenz et al., 2019; Moyle et al., 2019). Our analysis also shows some limitations in the existing AT-related literature. The first one is concerned with using heterogeneous methodologies to assess the impact of the use of ATs. Specifically, several devices deliver different types of interventions, the sample size is usually small, research designs barely standardized, and the outcomes were not enough systematized. Unfortunately, such limitations have not improved with time. Both heterogeneity and effect sizes featuring the latest studies showed that technologies need more controlled research to reveal their effectiveness. Furthermore, our analysis shows a possible bias regarding the population defined as the interventions' primary target. For example, many of the interventions monitoring PWD to offering them security services are often described as helpful “for caregivers.” Even though these technologies may also assist the caregiving process, we think that they should be labeled as “for PWD,” as the actual label does not consider functional autonomy levels still active. The security devices can be used autonomously by PWD until their autonomy has not yet been severely compromised. In this vein, Lorenz et al. (2019) reported a meaningful blog post written by a person with dementia. The post describes how he felt about the transition between the active and passive roles in domestic alarms management. In the first phase of the disease, notifications supported a person's autonomy until he could recognize the different sounds. Later, due to the disease's progression, the person could not understand the source of the sounds anymore. Therefore, when a caregiver's assistance becomes necessary to manage the technological devices, it may be more appropriate to label the technology as “for caregivers”: it cannot support any longer the PWD but, instead, the caregiver.

Besides what was discussed, it is also relevant to mention that the theoretical models underpinning technological offers need improvement. Only the review of Rathnayake et al. (2019) highlighted the theoretical backgrounds upon which the designing process of the Apps was based. On top of this, just two of the studies reviewed by them reported a theoretical model. This evidence confirms the need to increase the number of studies on technology that bases their hypothesis on theoretical models. Such an improvement would impact the rate of studies reproducibility, and it can also foster the capability of the research to disentangle which factors cause the observed effects (Kennelly, 2011). These limitations are associated with two consequences. Firstly, the single outcomes featuring each intervention are hard to disentangle and to generalize. Secondly, there is a significant gap between the theory underpinning the research and the implementation of the devices in everyday life, which is due to a lack of attention toward time-related factors and organizational determinants (Christie et al., 2018). Indeed, the inadequate follow-ups and insufficient consideration of the person's ongoing adaptation process provoke an over-time decline in the usage of ATs in the post-trial phases (Christie et al., 2018). Finally, the design of interventions should focus more on developing user-friendly technologies that can be personalized and updated by respecting users' evolving needs. Additionally, there is a limited interest in innovation supplied by national and local health organizations, mainly when the elderly are the target of the technology (Christie et al., 2018). Hence, it is necessary to regularly update the research to develop interventions able to exploit the maximal potential of modern technologies and supportive organizational plans aimed at overcoming the barriers experienced by healthcare professionals and the devices' final users (Meiland et al., 2017).

## Limitations

One of the limitations affecting the present work consists of the use of a heterogeneous methodology chosen. Specifically, to timely respond to the pandemic, we primarily decided to include in this work reviews of reviews. As we noticed that no review discussed the recent outbreak, we welcomed new studies reporting how the technology can support both PWD and caregivers. The second main limitation regards the selection criteria since no we did not analyze any gray literature sources. Although such a decision might have prevented additional evidence from emerging, it secured certified standards.

## Data Availability Statement

The original contributions presented in the study are included in the article/supplementary material, further inquiries can be directed to the corresponding author/s.

## Author Contributions

AP performed the literature search, outlined the results, and drafted the manuscript. RC discussed the search outcomes and supervised the process. IC and MV reviewed the manuscript. GO performed the literature search, discussed and reviewed the results, drafted the document, and managed the operations. All authors contributed to the article and approved the submitted version.

## Conflict of Interest

The authors declare that the research was conducted in the absence of any commercial or financial relationships that could be construed as a potential conflict of interest.
